# Polymer Coating Enabled Carrier Modulation for Single-Walled Carbon Nanotube Network Inverters and Antiambipolar Transistors

**DOI:** 10.3390/nano14181477

**Published:** 2024-09-11

**Authors:** Zhao Li, Jenner H. L. Ngai, Jianfu Ding

**Affiliations:** Security and Disruptive Technologies Portfolio, National Research Council Canada, 1200 Montreal Road, Ottawa, ON K1A 0R6, Canada; jennerholoong.ngai@nrc-cnrc.gc.ca (J.H.L.N.); jianfu.ding@nrc-cnrc.gc.ca (J.D.)

**Keywords:** carbon nanotubes, transistors, conjugated polymers, thin films, network

## Abstract

The control of the performance of single-walled carbon nanotube (SWCNT) random network-based transistors is of critical importance for their applications in electronic devices, such as complementary metal oxide semiconducting (CMOS)-based logics. In ambient conditions, SWCNTs are heavily p-doped by the H_2_O/O_2_ redox couple, and most doping processes have to counteract this effect, which usually leads to broadened hysteresis and poor stability. In this work, we coated an SWCNT network with various common polymers and compared their thin-film transistors’ (TFTs’) performance in a nitrogen-filled glove box. It was found that all polymer coatings will decrease the hysteresis of these transistors due to the partial removal of charge trapping sites and also provide the stable control of the doping level of the SWCNT network. Counter-intuitively, polymers with electron-withdrawing functional groups lead to a dramatically enhanced n-branch in their transfer curve. Specifically, SWCNT TFTs with poly (vinylidene fluoride) coating show an n-type mobility up to 61 cm^2^/Vs, with a decent on/off ratio and small hysteresis. The inverters constructed by connecting two ambipolar TFTs demonstrate high gain but with certain voltage loss. P-type or n-type doping from polymer coating layers could suppress unnecessary n- or p-branches, shift the threshold voltage and optimize the performance of these inverters to realize rail-to-rail switching. Similar devices also demonstrate interesting antiambipolar performance with tunable on and off voltage when tested in a different configuration.

## 1. Introduction

Single-walled carbon nanotubes (SWCNTs) have been shown to be promising semiconducting materials for future electronic applications [1]. For high-end applications, they could be aligned and closely packed to build short-channel field-effect transistors for high-performance computer chips, as a potential candidate to replace silicon-based electronics. For low-end applications, they could be deposited as random networks to construct thin-film transistors (TFTs) for display backplanes, the internet of things or chemical and biomedical sensors. Their solution processibility and intrinsic flexibility make them particularly attractive for cost-effective, printed electronics [2].

The control of the operation mode, such as p-type or n-type SWCNT-based TFTs, is essential for low-energy consumption electronics, such as CMOS logics. In ambient conditions, SWCNTs will be heavily p-doped by adsorbed moisture and oxygen; then, the n-branch will be totally suppressed [3]. The H_2_O and O_2_ redox couple could also lead to large hysteresis in their transfer curve, especially on hydrophilic dielectrics [4]. There are many n-doping strategies to counteract this p-doping effect from ambient air [5], using solutions of small molecules with strong electron-donating properties [6,7], crown-ether [8], bis(trifluoromethanesulfonyl)imide [9,10], etc. However, these doping approaches usually show poor stability, especially at higher temperatures, due to the decomposition or mobile nature of small molecules. In addition, the hysteresis could be even worse as the H_2_O/O_2_ redox couple still exists in the system. All these drawbacks could severely limit the practical applications of SWCNT-based TFTs in printed electronics.

Coating or encapsulating the SWCNT network with a polymer layer has also been demonstrated to be an efficient method to control the doping level of these TFTs [11]. Compared with small molecules, the polymer coating approach not only shows better stability, but it is also compatible with the printing process. Coatings from a mixture of water-soluble polymers have been utilized to control the threshold voltage of SWCNT network TFTs [12]. However, previous studies were mostly performed in air; thus, the doping effect of H_2_O/O_2_ could not be removed, and most TFTs still showed large hysteresis in their transfer curve [11,12]. 

In this work, we compared the performance of SWCNT network-based TFTs covered with some common polymer coatings. As all the tests were carried out in a glove box filled with nitrogen, the interference of ambient air was largely removed. Surprisingly, we found that most polymer layers will lead to n-doping, and the doping level is dependent on the electron-withdrawing strength of the functional groups. The well-controlled threshold voltage shift could be utilized to optimize the inverter performance and realize antiambipolar functionality by connecting two transistors together.

## 2. Experimental Section

### 2.1. Materials and Instruments

A Hitachi SU-5000 SEM instrument was used to obtain a SEM image of a random SWCNT network on a SiO_2_/Si substrate, with charge contrast imaging mode and 1 kV operation voltage. A Keysight Technologies B2902A source-measurement unit was used for TFT characterization. SWCNT/ poly(9,9′-didocecylfluorene) (PFDD) dispersion was enriched from Plasma touch raw soot (Raymor Nanointegris, with diameter centered at ~1.3 nm) following a previous reported procedure [13]. Polyacrylonitrile (Product No. 181315, average M_w_ 150,000), poly(vinylidene fluoride) (Product No. 182702, average M_w_ ~534,000 by GPC), Poly(methyl methacrylate-co-ethyl acrylate) (Product No. 182249, ethyl acrylate < 5 wt%, average M_n_ ~39,500 by GPC), poly(vinyl chloride) (Product No. 388270, inherent viscosity 1.24), polystyrene (Product No. 331651, average M_w_ 35,000), Poly(vinyl formal) (Product No. 182680, T_g_ 108 °C) and poly(acrylic acid) solution (Product No. 416002, average M_w_ ~250,000, 35% wt.% in H_2_O) were purchased from Sigma Aldrich Canada. Microposit^TM^ S1813^TM^ G2 positive photoresist was purchased from Dupont Canada. All other chemicals used were purchased from Sigma Aldrich Canada and used as received.

### 2.2. TFT Fabrication Process and Characterization

TFT chips with pre-patterned electrodes (Fraunhofer Germany, Gen 5, 230 nm thick SiO_2_ dielectric layer) were rinsed with acetone, soaked in 5% Hellmanex solution (10 min, 50 °C) and rinsed with water and isopropanol before being blow-dried with nitrogen. The chips were then soaked in PFDD/sc-SWCNT solution (25 mg/L, polymer/tube weight ratio 3/1) for 10 min before being rinsed with toluene. The chips were then transferred to a nitrogen-filled glove box and annealed at 200 °C for 1 h before characterization. Polymer solutions (5% in dry DMAc) were then drop-casted on the finger digitated channel of the TFT, before baking at 50 °C for 30 min to evaporate the solvent and annealing at 200 °C for 1 h in the glove box. TFTs with a channel length of 20 or 10 µm and a channel width of 2000 µm were used here. All TFT characterization was performed in the glove box, and V_G_ was swept at 0.12 V/s for the transfer curve test. The mobility was calculated from the linear regime of the transfer curve, following a parallel plate model with 15 nF/cm^2^ gate dielectric capacitance. The hysteresis was the difference in V_G_ from the two sweeping directions at half of the maximum source-drain current which was extracted from the transfer curve. To connect two TFTs, a silver metal conducting line was printed between their electrode pads using silver ink (Novacentrix JS-A291, 40 wt% Ag ink) with Microplotter II from Sonoplot. Silver ink was deposited onto the TFT substrate using a ceramic-based dispenser with a 100 µm nozzle diameter at a 0.3 V ultrasonic power level. The printed device was then annealed at 200 °C in a N_2_-filled glove box to sinter the silver ink into silver metal to create the bridging silver connection. For inverters, the connecting point was served as an out electrode and the common gate as an in electrode, the drain of one TFT and the source of the other as V_DD_ and V_SS_. To form an antiambipolar transistor, the same device connection as the invertor was used but the connected TFTs were tested in a different configuration. Their common gate, the drain of one TFT and the source of the other were used as V_G_, S and D for the antiambipolar transistor. 

## 3. Results and Discussion

**Figure 1:**
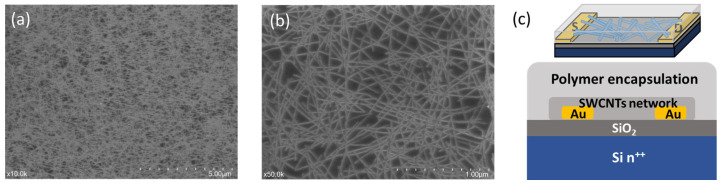
SEM images of SWCNT random network on SiO_2_ substrate at (**a**) low and (**b**) high magnification; (**c**) schematic illustration of TFT device structure.

High-semiconducting purity SWCNTs were extracted from a commercial plasma torched raw material following a previously established conjugated polymer purification process [13]. The diameters of the tubes are centered at 1.3 nm, and the average tube length is close to 0.9 µm. The final solution was adjusted to a tube concentration of ~25 mg/L and PFDD to a tube weight ratio of about 3/1. The tube solution was then deposited on a fresh cleaned silicon wafer with pre-patterned electrodes by a soaking and rinsing process, and a random-oriented but homogeneous tube network formed with a tube density of ~25 tubes/µm (Figure 1a,b). The weight ratio of the remaining polymer/tube should be less than 0.7/1, which could be easily obtained when the tube polymer solution was filtered on a PTFE membrane, where the tube layer could be much denser and thicker. After annealing at 200 °C for over 1 h in a glove box to enhance the tube adhesion and remove absorbed moisture and oxygen, polymer solutions at 5% in DMAc were drop-casted on the tube network to fully cover the active channel before slow baking to remove solvents and final annealing at 200 °C for 1 h in a glove box. It should be noted that after annealing at 200 °C, the SWCNT will strongly attach on the substrate, and the polymer solution drop-casting process will not change tube network morphologies, as demonstrated in the following device characterization part. The final device structure is shown in Figure 1c, and the SWCNT random network forms an active semiconducting channel on a SiO_2_ dielectric with a global back gate and bottom contacts.

**Figure 2:**
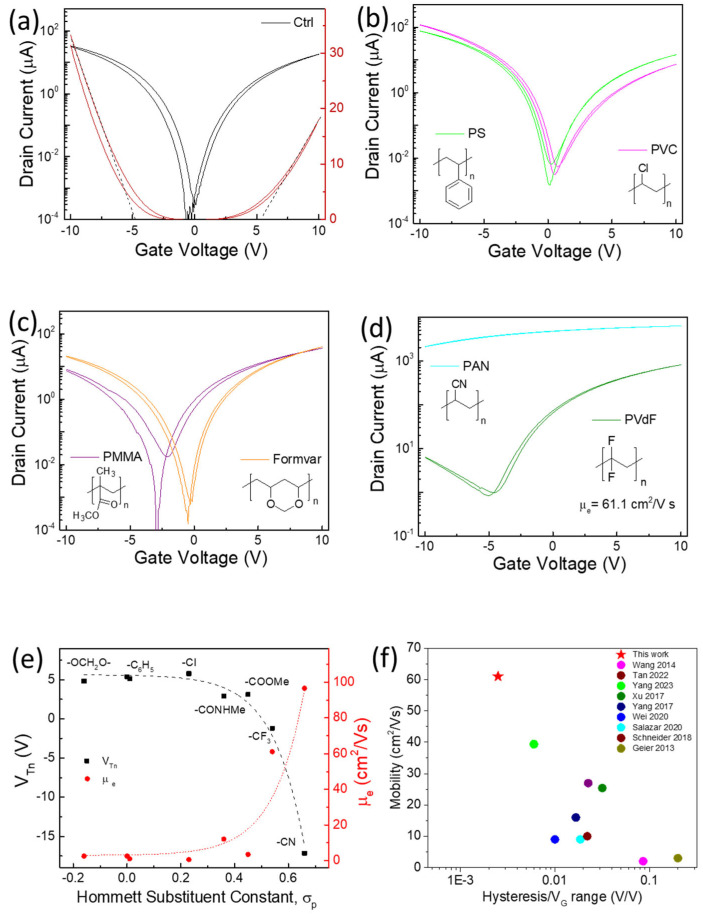
Representative transfer curves of SWCNT random network TFTs: (**a**) Ctrl, (**b**) coated with PS and PVC, (**c**) coated with PMMA or Formvar, (**d**) coated with PVdF or PAN; (**e**) extracted threshold voltage for n-branch (V_Tn_) and corresponding electron mobility (µ_e_) vs. Hommett substituent constant of structure similar functional groups within polymer; (**f**) comparison of mobility and hysteresis (normalized by V_G_ sweeping range) from PVdF-coated n-type CNT TFTs in this work with other reported random network CNT TFTs [6,10,20,21,22,23,24,25,26].

The representative device transfer curves are shown in Figure 2, and the TFT performance parameters are summarized in Table 1. SWCNT TFTs without polymer coatings (named as Ctrl hereafter) demonstrated quite balanced ambipolar behaviour (Figure 2a), with a large hysteresis of 0.62 V for hole transport and 0.26 V for electron transport within the gate sweeping range of ±10 V. Here, the hysteresis was defined as the difference in gate voltage (V_G_) between two sweeping directions at half of the maximum source-drain current. Comparatively, most polymer-coated TFTs showed decreased hysteresis (by 30–80%) due to the suppression of trapping sites on the SiO_2_ substrate [14] or redox active defects from CNT broken ends [15]. Coatings from polymers with electron-rich groups, such as polystyrene (PS) and poly vinyl chloride (PVC), slightly shifted the threshold voltage (V_T_) toward the positive side (Figure 2b). Polymers containing ester or vinyl formal groups, such as PMMA or Formvar, enhanced the n-branch and slightly shifted the threshold voltage to the negative direction (Figure 2c). More interestingly, polymers with strong electron-withdrawing groups, such as poly (vinylidene fluoride) (PVdF) or polyacrylonitrile (PAN), demonstrated an excellent or degenerated n-doping effect (Figure 2d). Specifically, PVdF-covered CNT TFTs showed electron mobility up to 61.1 cm^2^/Vs, with a very low hysteresis at 0.05 V for the n-branch and an on/off ratio at ~10^3^.

As shown in Figure 2e, there is a good correlation between the V_T_ of the n-branch with the Hommett substituent constant of the functional groups within the polymer [16], which was often used to characterize the electronic effect of a substituent on organic reactions [17]. A previous study has demonstrated that the ratio of pyridine units within a wrapping copolymer of polyfluorene is closely related to the V_T_ of CNT TFTs [18], where the weight ratio of the wrapping polymer to the CNT is only close to 1/1. In this work, the excess coating polymer would fully cover around tubes, and thermal annealing should further enhance their close contact as 200 °C is higher than the glass transition temperature of most of these polymers. The abundant functional groups near tubes could form a screening or field effect [19], and spatial charge transfer could happen for groups with strong electron-withdrawing or -donating capabilities. For PVdF or PAN, electron-withdrawing groups in the coating polymer can stabilize mobile electrons and thus shift the Fermi level towards the conduction band, which will enhance the n-doping level. Figure 2f compares both the electron mobilities and hysteresis widths of PVdF-covered SWCNT TFTs from this work with those reported in previous studies that feature random SWCNT network-based n-type TFTs [20,21,22,23,24,25,26]. For a reasonable comparison, the hysteresis widths were normalized to the V_G_ sweeping range. Clearly, our PVdF-coated SWCNT TFTs demonstrate both high mobility and low hysteresis compared with those in other studies.

**Figure 3:**
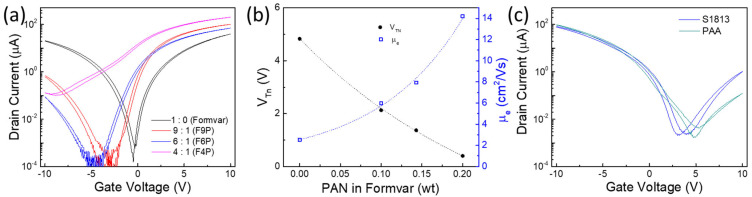
(**a**) Representative transfer curves from blends of Formvar and PAN-covered SWCNT network TFTs. (**b**) Threshold voltage and electron mobility vs. weight percentage of PAN within Formvar. (**c**) P-type transfer curves from PAA or photoresistor S1813-covered SWCNT network TFTs.

Formvar resin has been widely used for wire insulation, and Formvar coated SWCNT TFTs show balanced ambipolar charge transport with low hysteresis and high on/off ratio (Figure 2c). These properties make Formvar particularly attractive to be blended with other stronger n-doping polymers, such as PAN, to finetune the n-doping capability. As shown in Figure 3a, a mixed coating containing both PAN and Formvar could be used to gradually improve n-branch transport and still maintain a low hysteresis level. Here, the blends were named after the weight ratio of Formvar to PAN; for example, F6P represents a blend with a Formvar-to-PAN weight ratio of 6/1. As the weight of PAN increased from 0 to 20% (F4P) in the blends, V_T_ for the n-branch shifted from ~5 V to 0.5 V, while the electron mobility increased from 2.5 to 14 cm^2^/Vs (Figure 3b). 

Most other common polymers tested, such as polyvinyl pyrrolidone, Teflon-AF and cellulose acetate, only slightly shift the transfer curve with the change in V_Th_, and µ_e_ falls within the range of that in Figure 2e [22,27]. Only polyacrylic acid (PAA) and a positive photoresist S1813 can effectively suppress an n-branch but with the sacrifice of slightly increased hysteresis (Figure 3c) [28]. Hereafter, the polymer-covered CNT TFTs are designated with the abbreviation of the coating polymers in the device diagram.

**Figure 4:**
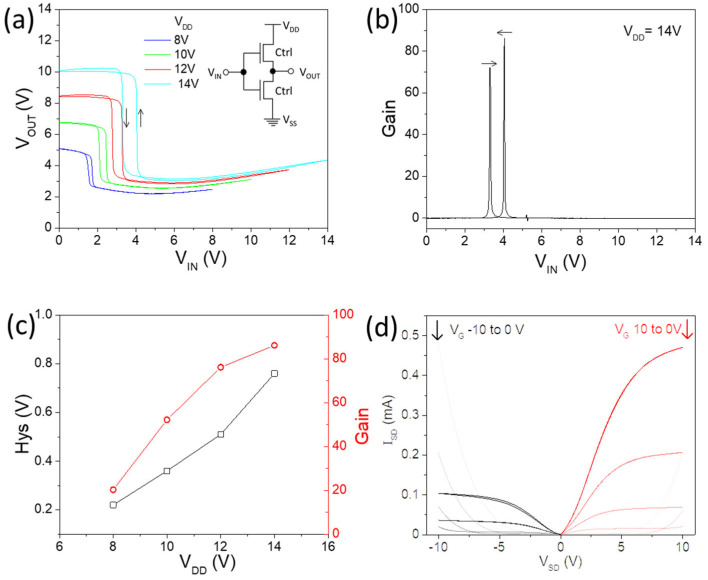
(**a**) Voltage transfer characteristics of inverter by connecting two ambipolar Ctrl TFTs; inset is circuit diagram; (**b**) gain at V_DD_ of 14V; (**c**) hysteresis and gain vs. V_DD_; (**d**) output characteristics of representative Ctrl TFT.

The ambipolar characteristics of the Ctrl TFTs make it possible to build an inverter, the fundamental component of integrated circuits, by simply connecting two TFTs with printed silver lines. As shown in Figure 4a, the inverter demonstrates typical ‘Z’-shaped voltage transfer characteristics (VTCs), with high gain up to ~80 at a V_DD_ of 14 V, which could be useful as a biosignal amplifier [29]. There is severe voltage loss at both the high- and low-input voltage sides, because the two ambipolar TFTs behave very similarly. When one TFT is turned on and the other cannot be completely switched off [30], the hysteresis increased with higher V_DD_ (Figure 4c), because a wider V_G_ sweep range will lead to severer hysteresis for each single TFT. In addition, the switching threshold is not located near V_DD_/2 because of the mismatch between the p/n-branch, as shown in the output characteristics (Figure 4d).

**Figure 5:**
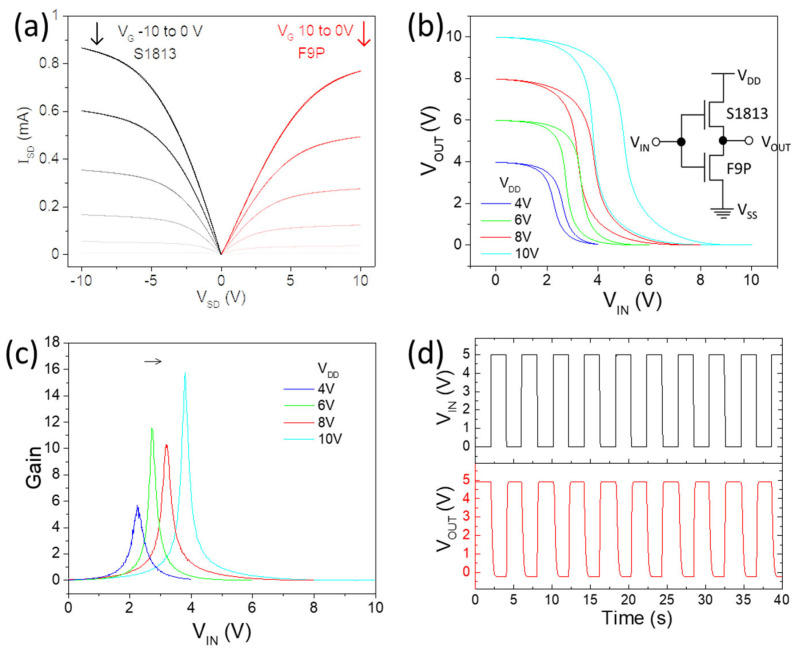
(**a**) Output characteristics of p-type and n-type polymer-coated CNT TFTs; (**b**) voltage transfer characteristics of inverter by connecting p/n-type TFTs, with inserted circuit diagram; (**c**) gain at various V_DD_; (**d**) input and output waveforms of inverter operated at 5 V.

The polymer coating approach developed above enabled us to effectively adjust the p/n-doping level. One of the Ctrl TFTs was covered by S1813 to suppress the n-branch, and the other was coated with F9P to enhance it; then, balanced output characteristics can be realized (Figure 5a). The inverter shows rail-to-rail VTCs (Figure 5b), and the switching threshold is close to V_DD_/2. The large hysteresis mainly comes from p-type TFTs, which were covered by photoresist S1813. There is a trend that a higher V_DD_ will give better gain (Figure 5c), although the gain of ~11 at a V_DD_ of 6 V is slightly higher than the gain of ~10 at a V_DD_ of 8 V. Figure 5d shows the input and output waveform of the inverter working at a V_DD_ of 5 V, which demonstrates well-controlled and repeatable switching.

Recently, antiambipolar transistors (AATs) have attracted much attention, in which the drain current can be turned on within a certain range of the gate bias [31]. Usually, the active channel of an AAT consists of partially overlapped p/n-type materials, and their hetero-interface is of critical importance for proper function. CNT network channels have been patterned and partially covered with PMMA and polyethylenimine to prepare p-n diodes [32]. CNT networks have also been combined with other materials, such as MoS_2_ [33] and amorphous indium gallium zinc oxide (IGZO) [34], to construct p-n heterojunctions with antiambipolar transfer characteristics. 

**Figure 6:**
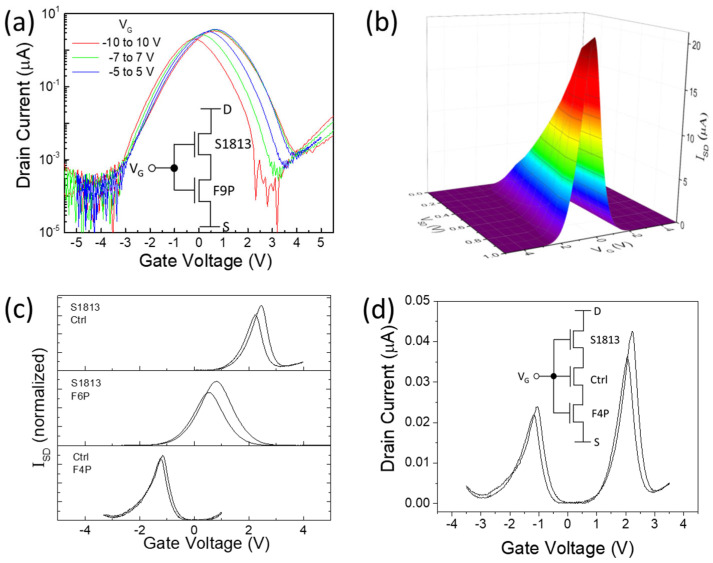
(**a**) Drain current—V_G_ curves of the antiambipolar transistor by connecting two SWCNT TFTs with different polymer coating layers; the circuit diagram is shown in the inset; (**b**) a 3D plot of the drain current depending on both drain and gate bias voltage; (**c**) normalized drain current—V_G_ curves of three antiambipolar transistors with finetuned doping levels for each TFT, with the coating polymers for each AAT shown on the left; (**d**) drain current—V_G_ curves of a double antiambipolar transistor by connecting three SWCNT TFTs with different doping levels.

There is another recently reported method to realize similar “Λ-shaped” transfer curves as in AATs by simply connecting two p- and n-type TFTs together [35]. Compared with AATs, this new structure avoids the interface of the p/n-junction, and shoot-through current is the main working mechanism, as in conventional CMOS devices [36]. Figure 6a shows the Λ-shaped transfer curve from the same device used before for inverters but with different connections for AAT testing (inset in Figure 6a). The driving voltage to yield the highest peak current (3.4 µA) is near 0 V, with a high peak-to-valley ratio (PVR) of ~2 × 10^4^. The on-set and off-set voltage are at −4 and 3 V, with a driving range of about 7 V. The hysteresis between forward and backward sweep is the combination of individual TFTs, which also depends on the sweeping range. Similar to other AATs, the peak current demonstrates a linear relationship with the source-drain voltage (Figure 6b). The well-controlled threshold voltage of individual TFTs from different polymer coatings enables us to effectively adjust both the on-set and off-set voltage of the resulting AATs, as shown in Figure 6c. In addition, Figure 6d demonstrates a double Λ-shaped transfer curve by connecting three TFTs with different doping levels, where the drain current could be turned on at two different ranges of gate bias voltage. This double antiambipolar transistor could be particularly attractive for multivalued logic applications [37].

There are several distinct advantages for this kind of AAT. First, only high-mobility CNT networks have been used as active channels; thus, any potential compatibility problem from the heterojunction interface between two different materials could be avoided. Second, this polymer coating approach enables a quite wide range of well-controlled V_T_, corresponding to tunable on and off voltages for AATs. Third, both the active semiconducting SWCNT and coating polymer materials are intrinsically flexible. Forth, the simple solution process could be particularly attractive for low-cost, disposable, wearable and flexible electronic applications [31].

## 4. Conclusions

In summary, we developed a simple doping control strategy by covering the random SWCNT network with common polymer materials. Tested in a nitrogen-filled glove box, the p-doping effect from an ambient atmosphere was largely removed, and the intrinsic doping capabilities of polymer coating layers on SWCNT TFTs were revealed. It was found that the p/n-doping level is closely related to the electron-donating or -withdrawing properties of the functional groups within the polymer, while most polymer coating layers will reduce the hysteresis of these TFT devices due to the partial removal of trapping sites. Polymers with electron-rich groups, such as PS and PVC, will slightly p-dope tubes. PMMA and Formvar will slightly n-dope tubes, as they contain ester or ether groups. PVdF, which contains strong electron-withdrawing groups, demonstrated an excellent n-type doping effect, with an electron mobility of up to 61 cm^2^/Vs and a very low hysteresis of 0.05 V at the gate sweeping range of ±10 V. Blends from two polymers with different doping capabilities could be used to finetune the n-doping level. This polymer coating doping approach enabled SWCNT TFT-based inverters with rail-to-rail performance and realized interesting antiambipolar transistor behaviour. We hope that this solution processible polymer doping method can broaden the road towards the commercialization of SWCNT TFT-based printable and flexible electronics.

## Figures and Tables

**Table 1:** Device performance of pristine (Ctrl) and polymer-coated SWCNT TFTs.

Coating Polymers	p-Branch (Hole Transport)				n-Branch (Electron Transport)			
	µ_h_^a)^ [cm^2^/Vs]	V_Th_ [V]	On/Off ^b)^	Hysteresis ^c)^ [V]	µ_e_ ^a)^ [cm^2^/Vs]	V_Te_ [V]	On/Off ^b)^	Hysteresis ^c)^ [V]
Ctrl	4.22 ± 0.28	−4.85 ± 0.07	10^5.6 ± 0.1^	0.62 ± 0.05	2.47 ± 0.10	5.24 ± 0.11	10^5.4 ± 0.1^	0.26 ± 0.05
PS	4.67 ± 0.22	−4.56 ± 0.06	10^4.7 ± 0.1^	0.16 ± 0.02	0.99 ± 0.12	5.15 ± 0.08	10^4.0 ± 0.1^	0.05 ± 0.02
PVC	7.63 ± 0.24	−4.89 ± 0.06	10^4.6 ± 0.1^	0.21 ± 0.03	0.58 ± 0.08	5.78 ± 0.12	10^3.4 ± 0.1^	0.08 ± 0.03
PMMA	1.34 ± 0.29	−6.07 ± 0.10	10^5.0 ± 0.1^	0.46 ± 0.06	3.59 ± 0.12	3.31 ± 0.22	10^5.7 ± 0.1^	0.20 ± 0.06
Formvar	1.42 ± 0.22	−5.15 ± 0.05	10^5.1 ± 0.1^	0.32 ± 0.04	2.53 ± 0.07	4.82 ± 0.12	10^5.4 ± 0.1^	0.11 ± 0.04
PVdF	0.96 ± 0.23	−5.82 ± 0.08	10^0.9 ± 0.1^	0.24 ± 0.05	61.1 ± 0.22	1.26 ± 0.12	10^3.0 ± 0.1^	0.05 ± 0.02
PAN	-	-	-	-	96.7 ± 1.54	−17.24 ± 1.35	-	0.36 ± 0.10

^a)^ The average mobilities were calculated from 4 TFT devices. ^b)^ On/off was calculated from the ratio of the highest and lowest current in the transfer curve within the sweeping range of V_G_ between −10 and 10 V at a V_SD_ of 1V. ^c)^ Hysteresis was defined as the V_G_ difference from the forward and reverse sweeping directions at half of the highest source-drain current.

## Data Availability

Data are contained within the article.
